# Editorial: CRISPR-Cas Systems in Bacteria and Archaea

**DOI:** 10.3389/fmicb.2022.887778

**Published:** 2022-03-29

**Authors:** Muhammad Kamruzzaman, Aixin Yan, Graciela Castro-Escarpulli

**Affiliations:** ^1^Centre for Infectious Diseases and Microbiology, Westmead Institute for Medical Research, The University of Sydney, Sydney, NSW, Australia; ^2^School of Biological Sciences, The University of Hong Kong, Pokfulam, Hong Kong SAR, China; ^3^Escuela Nacional de Ciencias Biológicas, Instituto Politécnico Nacional de México (IPN), Mexico City, Mexico

**Keywords:** CRISPR-Cas, adaptive immunity, spacers, gene editing, bioethics

The CRISPR-Cas [clustered regularly interspaced short palindromic repeats and the CRISPR-associated genes (Cas)] is an adaptive immune system of prokaryotes against the invasion of foreign genetic elements and is widely distributed in the chromosomes of most archaea and many bacteria (Garneau et al., 2010; Marraffini, 2015; Hille et al., 2018). The system consists of a CRISPR array, comprising of short direct repeats, separated by short variable DNA sequences (called “spacers”) acquired from foreign genetic elements and is flanked by various Cas genes. Cas genes are highly diverse and are involved in the different stages of CRISPR activity. Even though CRISPR-Cas is known as a defense system of prokaryotes, they are involved in different non-defense roles, including bacterial biofilm formation, regulation of quorum sensing, and pathogenicity. This special issue aims to collect articles that shed light on the recent advances in the CRISPR-Cas research to better understand the distribution, diversity, and biological functions of CRISPR-Cas systems. We have collected nine articles that highlight the recent studies on distribution, structure, biological functions and applications of CRISPR-Cas, as well as ethical considerations of CRISPR-Cas research.

Bioinformatic analysis of 716 genomes of *Staphylococcus aureus* (by Cruz-López et al.) identified that only 0.83% of *S. aureus* strains of the different geographical regions have type IIA CRISPR-Cas system, suggesting the occurrence of CRISPR-Cas in *S. aureus* may be spontaneous horizontal gene transfer event. 0.9% of the unique spacers matched with either plasmid or phage genomes, including bacteriophages used for the therapy against *S. aureus* infection, indicating the development of phage resistance *S. aureus* and therapeutic failure due to the CRISPR defense mechanism.

Direct uptake of foreign DNA from surrounding environments plays an important role in the genome diversity and evolution in bacteria and archaea. Liu et al. reviewed the functions and possible mechanism of the CRISPR systems and Argonauts in cellular defense against natural transformation. A limited number of studies demonstrated that type II CRISPR-Cas could prevent natural transformation in bacteria; however, the exact mechanism and whether other types of CRISPR systems also antagonize natural transformation is not known. Argonauts also can prevent the natural transformation of plasmid DNA. Unlike CRISPR-Cas systems, argonauts-mediated defenses do not integrate DNA fragments into host genomes and, thus, no memory of the invading DNA is generated.

To optimize sequence-specific immunity against invading genetic elements, CRISPR-Cas in prokaryotes continuously acquire spacers from the newly invading threats. Over time, many of the acquired spacers may become useless in their defense mechanism. Therefore, spacer uptake, their existence and loss must be regulated. A very interesting review by Garret compiled different observations and experimental designs to speculate a model for the spacer dynamics in the CRISPR array and demonstrated that new spacers are added at the leader end of the array, which varies among species, systems, and conditions. Rearrangement of the array is ongoing at some level, though the particular frequency is also variable among species and CRISPR-Cas classes. The terminal spacer-repeat unit rarely participates in rearrangements, so the array is maintained, and the last spacer-repeat unit is stable.

Type IV CRISPR-Cas system, primarily found on plasmids (Kamruzzaman and Iredell, 2019), is least understood among the six CRISPR types. The lack of Cas nucleases, integrases, and other genetic features commonly found in most CRISPR systems has made it difficult to predict the mechanisms of action and biological functions of type IV CRISPR-Cas. The perspective by Taylor et al. compiled and analyzed recent advances in bioinformatics, biochemical, and structural studies of type IV systems that provided valuable insights to understand the structure and function of type IV systems. Instead of Cas gene *csf1*, Cas-7 like gene *csf2* was proposed to be employed to distinguish type IV from other types in the Class1 CRISPR-Cas group. Type IV-A systems protect bacteria from plasmids and phages, which needs DinG helicase along with other Cas proteins with an unknown mechanism of action. Recently identified type IV-C systems lack a Csf1 subunit and instead encode a Cas10-like subunit with an HD nuclease Domain, while type IV-B systems lack a CRISPR locus and a crRNA processing enzyme and are associated with an ancillary gene identified as cysH-like. The mechanism of action and biological functions of type IV-B and -C are yet unknown.

Type III CRISPR-Cas systems can target both RNA and single-stranded DNA and provide immunity against invaders, which is dependent on the target RNA transcription. The target RNA binding also activates the cyclic oligoadenylate (cOA) synthesis activity of Cas10 subunit. The recent advances on cOA synthesis, cOA-activated effector protein, cOA signaling-mediated immunoprotection, cOA signaling inhibition, and possible crosstalk between cOA signaling and other cyclic oligonucleotide-mediated immunity have been discussed in the review by Huang and Zhu.

CRISPR-Cas is not only involved in bacterial defense mechanisms but also involved in the regulation of bacterial physiology. One example is the very conserved CRISPR-Cas system found in the *Salmonella* Typhi, which regulates the synthesis of major outer membrane proteins (OMP) OmpC, OmpF, OmpA and quiescent OMP, OmpS2 by regulating the expression of the master porin regulator OmpR (Medina-Aparicio et al.). This CRISPR-Cas system is also involved in the resistance to bile salts and in the formation of biofilms by *Salmonella* Typhi.

The application of CRISPR-Cas in genome editing for both prokaryotes and eukaryotes revolutionized genetic engineering technology. CRISPR-Cas gene-editing tool has enormous potential as antimicrobial agents, and Wang et al. successfully eliminated two virulence plasmids from *Bacillus anthracis* and *B. cereus* and specifically killed *B. anthracis* using the CRISPR/Cas9 system. The nuclease activity of CRISPR-Cas protein allows researchers to edit a genome with unprecedented ease, accuracy, and high throughput, while CRISPR interference (CRISPRi) technology has been developed for silencing specific genes by exploiting the catalytically inactive Cas9 (dCas9) and single-guide RNA (sgRNA). RNA interference (RNAi) technology is mainly used in eukaryotes to investigate the function of essential genes. The development of the CRISPRi system will provide a high-throughput, practical, and efficient tool for the discovery of functionally important genes in bacteria. The mini-review by Zhang et al. discussed the CRISPRi system, the underlying mechanism and properties and highlighted its application as a high-throughput screening tool in gene function analysis.

Finally, the precise gene editing capacity of CRISPR-Cas opens new possibilities to treat genetic diseases which are untreatable so far. But cautions need to be taken, and the processes need to be regulated to ensure patients' safety and implementation of bioethics (review by Gonzalez-Avila et al.). Scientists first need to understand the risk associated with any particular genetic modification, and CRISPR-based therapeutics must not be misprescribed or used for personal prejudices but always be approved by institutional and specialized bioethics committees. Scientists and world leaders should set boundaries about the use of CRISPR technologies.

## Author Contributions

MK prepared the call for this Research Topic. MK, AY, and GC-E edited the manuscript that were submitted. MK wrote this editorial. AY and GC-E reviewed it. All authors approved the submitted version.

## Funding

MK was supported by the Australian National Health and Medical Research Council (NHMRC) Grant (APP1197534) during the writing of this editorial.

## Conflict of Interest

The authors declare that the research was conducted in the absence of any commercial or financial relationships that could be construed as a potential conflict of interest.

## Publisher's Note

All claims expressed in this article are solely those of the authors and do not necessarily represent those of their affiliated organizations, or those of the publisher, the editors and the reviewers. Any product that may be evaluated in this article, or claim that may be made by its manufacturer, is not guaranteed or endorsed by the publisher.
